# The Accumulation of Abscisic Acid Increases the Innate Pool of Soluble Phenolics through Polyamine Metabolism in Rice Seedlings under Hexavalent Chromium Stress

**DOI:** 10.3390/toxics12080577

**Published:** 2024-08-08

**Authors:** Yi Kang, Cheng-Zhi Li, Abid Ullah, Qing Zhang, Xiao-Zhang Yu

**Affiliations:** College of Environmental Science & Engineering, Guilin University of Technology, Guilin 541004, China; 1020210405@glut.edu.cn (Y.K.); 1020190262@glut.edu.cn (C.-Z.L.); 2023009@glut.edu.cn (A.U.); zhangqing@glut.edu.cn (Q.Z.)

**Keywords:** abscisic acid, hexavalent chromium, rice, spermine, total soluble phenolics

## Abstract

Potential toxic element (PTE) pollution has emerged as a significant environmental and social concern in global agriculture. Chromium (Cr) occurs in different oxidation states naturally, among them Cr(VI), which is highly toxic. This study carried out biochemical and molecular tests to elucidate the accumulation of total soluble phenolics (TSPs) in rice plants exposed to Cr(VI) at 2.0, 8.0, and 16.0 mg Cr/L, emphasizing the interaction between polyamines (PAs) and abscisic acid (ABA). The results revealed significant Cr accumulation in different tissues of rice plants, which hindered their growth. Cr(VI) exposure increased the ABA concentration, with higher levels detected in the shoots than in the roots. The TSP concentration in rice tissues showed a positive relationship with the supplied concentrations of Cr(VI). The measured PAs, including spermine (Spm), putrescine (Put), and spermidine (Spd), exhibited varied responses to Cr(VI) stress, with only Spm concentration increasing with Cr(VI) concentrations. Real-time qRT-PCR showed PAs and ABA synthesis-associated genes such as *OsADC1*, *OsAIH*, *OsCPA1*, and *OsCPA4* were significantly up-regulated in shoot of rice plants treated with Cr(VI). These genes are associated with the second pathway of Put synthesis, originating from Arg. Almost all genes activated in the Met pathway were significantly up-regulated as well. Moreover, the genes involved in the interconversion among the three species of PAs exhibited completely different responses to Cr(VI) exposure. Overall, the biochemical analysis and gene expression data indicate that the interaction between ABA and Spm is likely to enhance the TSP levels in rice plants subjected to Cr(VI) toxicity.

## 1. Introduction

The term “potentially toxic elements” (PTEs) is often used to define a category of metals that are related to contamination and possible toxicity, which are strongly reliant on their doses and exposure period [1]. The impact of PTE contamination on global agriculture has been extensively recorded. Plants grown in contaminated soils have initiated various efforts to achieve a homeostasis between growth and defense, ultimately minimizing the damage caused by PTE [2]. One such effort involves the active transport and isolation of metal ions into the cell wall and other non-functional fractions, reducing the targeted attack by metal ions [3]. Another detoxification pathway involves the complexation of metal ions with diverse ligands or proteins to cope with PTE stress [4]. Additionally, plants are naturally equipped with cellular antioxidant systems to detoxify, scavenge, and neutralize additional reactive oxygen species (ROS) induced by PTE exposure through enzymatic reactions or non-enzymatic compounds [5,6]. Chromium (Cr) is the seventh most prevalent element in the Earth’s crust and is also recognized as one of the top 20 dangerous compounds on the Superfund priority list [7]. The frequent discharge of Cr from both natural and human activities has led to a significant increase in its contamination and distribution in the environment. The most prevalent species are Cr(VI) and Cr(III) detected in different environmental matrices [8], posing significant risk to living organisms, from individuals to ecosystem, due to their diverse toxic effects [6]. Cr(VI) is widely acknowledged as a hazardous chemical that has carcinogenic and mutagenic effects on humans. On the other hand, a tiny amount of Cr(III) is advantageous for the metabolism of sugar and lipids in living organisms as it acts as a micronutrient [6,7]. Cr(VI) and Cr(III) exhibit distinct characteristics in terms of their occurrence, chemical properties, environmental behavior, and toxicity [7]. Numerous studies have been undertaken to understand the potential mechanisms associated with the absorption, storage, transport, phytotoxicity, and detoxification of Cr(VI) in different tissues of plants [5,7,8,9,10]. For instance, Cr(VI) is bioavailable for plants, but variation in the capacity of uptake and distribution between plant species is highly dependent on genetic traits [10]. Moreover, oxidative damage resulting from higher levels of ROS in plants subjected to Cr(VI) stress causes disorder and/or dysfunction of redox balance and cellular homeostasis, restricting plant growth [7,8].

Under non-stress conditions, primary metabolites (PMs), secondary metabolites (SMs), and phytohormones are recognized to play crucial interconnected roles within the metabolic networks of plants [11]. Maintaining stable intracellular pools of these compounds is considered a prerequisite for normal plant functioning [11,12]. A variety of reactions and processes in plants are activated to regulate the intracellular balance through a signaling cascade during various abiotic stimuli [13,14,15]. The dynamic crosstalk and trade-offs between the key elements in the metabolic network are critical and depend on plant species, developmental phase, and the duration and types of stresses [12,13]. Secondary metabolites, which are non-nutritional bioactive substances derived from primary metabolism [16], are diverse and variable in plants, potentially serving as a bioindicator for stress management [17,18]. For instance, exposure to cadmium (Cd) and nickel (Ni) has been observed to increase phenolic metabolites, which serve as common indicators of nitrogen (N) deficiency in chamomile plants [19,20]. Besides SMs, polyamines (PAs), such as spermidine (Spd), spermine (Spm), and putrescine (Put), are derivatives of PMs that regulate plant growth and survival [21]. Evidence indicates that PAs function within a broad array of biological processes [22,23] and play multiple physiological roles in plants’ adaptive responses to a variety of stresses [24,25,26,27]. For instance, Cd treatment in wheat leaves increases endogenous Put levels due to the enhanced enzymatic functions of ornithine decarboxylase (ODC) and arginine decarboxylase (ADC) [28]. Many studies suggest that PA’s role in stress management is related to modulating ROS homeostasis by activating internal antioxidant systems or inhibiting ROS formation [25]. The overall response of plants to various stresses is governed by stress hormones [29]. Experimental evidence has confirmed that abscisic acid (ABA) serves as the primary mediator and signaling molecule activated in various abiotic conditions [30].

The reorientation of PAs triggered by ABA signals in grapevines subjected to drought stress under greenhouse conditions was evident, with the innate level of PAs being highly stimulated by the presence of ABA in the plants [21]. It is suggested that hormonal signaling pathways induce a metabolic shift from primary to secondary metabolic processes [31,32]. Additionally, the role of PAs as a signal regulator in plants under stress conditions has been proposed [28]. Extensive research has examined the damage of Cr(VI) toxicity in plants at phytochemical, biochemical, morphological, and molecular levels [4,6,7,8,9,33]. From these results, we hypothesized that the specific impact of the interactions between ABA and PAs on the changes in total soluble phenolics (TSPs) in rice plants subjected to Cr(VI) most likely exists, which would expand our understanding of the crosstalk in a plant’s metabolic network under stress conditions. In this regard, a hydroponic system was established with the specified objectives: (1) to assess the growth of rice plants treated with Cr(VI) and the distribution of Cr(VI) in their shoots and roots; (2) to measure the concentrations of TSPs, ABA, arginine (Arg), ornithine (Orn), methionine (Met), Put, Spd, and Spm in the shoots and roots of Cr(VI)-treated rice plants; and (3) to investigate the expression abundance of genes encoding arginase (ARG), ODC, ADC, spermidine synthase (SPDS), *S*-adenosylmethionine synthase (SAMS), N-carbamoylputrescine amidohydrolase (CPA), agmatine iminohydrolase (AIH), *S*-adenosylmethionine decarboxylase (SAMDC), spermine synthase (SPMS), 9-*cis*-epoxycarotenoid dioxygenase (NCED), zeaxanthin epoxidase (ZEP), and ABA 8’-hydroxylase (ABA8ox) in Cr(VI)-treated rice seedlings. This study seeks to shed light on how rice plants respond to Cr(VI) stress and improve our understanding of the metabolic interactions within plants.

## 2. Materials and Methods

### 2.1. Seedlings Preparation and Experiment Design

Rice seeds (*Oryza Sativa* L. XZX45), a regular medium-matured indica rice variety (*Oryza Sativa* L. XZX45 ZY903 ♀ × ZF504 ♂), were sourced from the Hunan Academy of Agricultural Sciences (Hunan, China) [6] and immersed in distilled water for a period of 24 h before being sown in river sand. Germination took place in a climate-controlled chamber maintained at 25 ± 0.5 °C and 65 ± 2% relative humidity. During the entire growth of the seedlings, a modified version of 8692 nutritional solution was used to maintain nutrients [34]. After 16 days, the seedlings were harvested and cleaned with an ionic removal buffer. Subsequently, these seedlings were exposed to 0, 2.0, 8.0, and 16.0 mg Cr/L of the prescribed nutrient solution for a 2-day period. Potassium chromate (K_2_CrO_4_) was used for Cr(VI) treatments. These concentrations correspond to the three effective Cr(VI) levels (EC_20_, EC_50_, and EC_75_) identified in previous research [33]. To minimize water loss and prevent algal growth, aluminum foil was warped to each flask. The experiments included four independent replicates, and all chemicals of analytical purity (AR) grade were purchased from Aladdin Chemistry, Co., Ltd. (Shanghai, China).

### 2.2. Growth Measurement of Rice Seedling

The growth response of rice seedlings to varying Cr(VI) concentrations was determined by measuring changes in fresh weight changes, as outlined in previous research [33].

### 2.3. Measurement of Cr

After the 2 days of treatment, Cr concentration in different tissues of rice plants was evaluated. The seedlings underwent a cleaning process using deionized water and were then divided into shoot and root tissues. The digestion solution (4:1 HNO_3_-HClO_4_) was used to digest the oven-dried plant materials (48 h at 96 °C). The concentration of Cr in rice tissues (μg/g DW) was measured with the help of ICA-AES (PerkinElmer Optima 700DV). For the element measurement, Cr at 267.716 nm was used. The detection limits, determined as mean blank plus three times the standard deviation of ten blanks, was 0.07 μg Cr/L.

### 2.4. Measurement of ABA

After exposing seedlings to Cr(VI), their tissues were harvested and subsequently ground, following the protocol outlined in Zhang et al. [33]. The resulting tissue powders were then utilized for isolating, refining, and analyzing ABA. The ABA (ng/g FW) concentration was quantified using ultra-performance liquid chromatography–tandem mass spectrometry (UPLC-MS/MS). Detailed information about the procedures for isolation, refinement, and analysis can be found in the Supplementary Materials.

### 2.5. Extraction and Quantification of Total Soluble Phenolics

The extraction process for TSPs is described in previous studies [19,35] with slight modifications. Following exposure, the seedlings underwent rinsing with distilled water and were separated into shoots and roots. Fresh plant tissues (0.3 g) were homogenized in 5 mL of 80% methanol while kept on ice. The homogenate was then centrifuged at 10,000× *g* for 5 min at 4 °C. The supernatant from the centrifugation was utilized for the measurement of TSPs. The concentration of TSPs in the supernatant was measured using the Folin–Ciocalteu method, detailed in the Supplementary Materials.

### 2.6. Measurement of Polyamine-Related Compounds

Following exposure, the shoots and roots of rice plants were ground in liquid nitrogen. The specific details of the sample preparation are documented in Zhang et al. [33]. Subsequently, the concentrations of Arg, Orn, Met, Put, Spd, and Spm (nmol/g FW) in the rice shoots and roots were quantified using the protocol outlined in the Supplementary Materials.

### 2.7. RNA Extraction and RT-qPCR Analysis

For the isolation of RNA from Cr(VI)-treated and untreated rice shoots, an Ultrapure RNA Kit (CWBio, Taizhou, China) was employed. To ensure the removal of any potential genomic DNA contamination, the RNA extracts underwent DNase I treatment. Following the DNase I treatment, the RNA was further purified using an RNeasy MinElute Cleanup Kit (Qiagen, Hilden, Germany) to enhance its purity.

A set of 30 genes encoding 8 enzymes related to PM metabolism and 3 enzymes related to ABA synthesis were selected for RT-qPCR analysis. These genes include ARG (1 gene), ODC (2 genes), ADC (3 genes), AIH (1 gene), CPA (4 genes), SAMS (4 genes), SAMDC (3 genes), SPDS/SPMS (3 genes), ZEP (1 gene), NCED (4 genes), and ABA8ox (3 genes). The specific primer sequences of these genes are listed in the Supporting information, Table S1.

The RT-qPCR experiments were conducted using the 7500 Fast system (Applied Biosystems) with SYBR green chemistry. The conditions were 95 °C for 10 s, 58 °C for 30 s, and 72 °C for 32 s, repeated 40 times. To ensure accurate normalization, the housekeeping gene *OsGAPDH* (LOC_Os08g03290.1) was used, as described by Yang et al. [36]. Relative gene expression levels were determined using the standard 2^−ΔΔCT^ method [37].

### 2.8. Data Analysis

The results are presented as mean ± SD. Statistical analysis was conducted at the 0.05 level of significance using SPSS software version 17.0 (SPSS, Inc., Chicago, IL, USA). Significant differences are indicated by the asterisk comparing treated seedlings to the control. To analyze the genetic subcellular localization, the Uniprot (https://www.uniprot.org/ accessed on 10 July 2024) program was utilized. The heat-map of gene expression was created with the Mev4.0 online program (MultiExperiment Viewer v.4.9.0).

## 3. Results

### 3.1. Growth Response of Rice Plants to Cr(VI)

**Figure 1:**
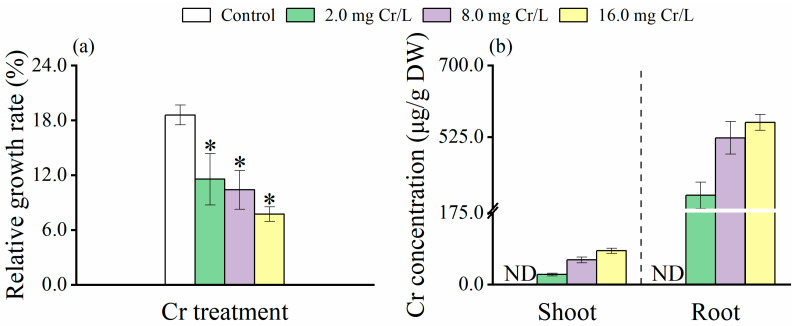
The relative growth rate (RGR %) (**a**) and total Cr concentration (μg/g DW) (**b**) in roots and shoots of rice seedlings under Cr(VI) stress. The control is 0.0 mg Cr/L. Values are the mean of four independent biological replicates ± standard deviation. ND denotes concentrations below the limit of Cr detection. The asterisk (*) refers to the significant difference between the treatments and the control.

The growth response of rice plants to various Cr(VI) concentrations was evaluated using their relative growth rate (RGR, %). As shown in Figure 1a, a significant reduction in RGR (*p* < 0.05) was detected with increasing concentrations of Cr(VI), highlighting the negative impact of higher Cr(VI) levels on seedling growth.

### 3.2. Distribution of Cr(VI) in Rice Tissues

The background levels of Cr (μg/g DW) in the shoots and roots of rice plants without Cr(VI) treatment were below the detection limit. In contrast, Cr(VI)-treated seedlings showed remarkably higher levels (*p* < 0.05) of Cr, with the accumulation rates increasing in a dose-dependent manner as the exogenous Cr(VI) concentrations rose (Figure 1b). It is also noticed that a significantly higher accumulation of Cr (*p* < 0.05) was observed in the roots compared to the shoots of Cr(VI)-treated seedlings.

### 3.3. Changes in ABA Level

**Figure 2:**
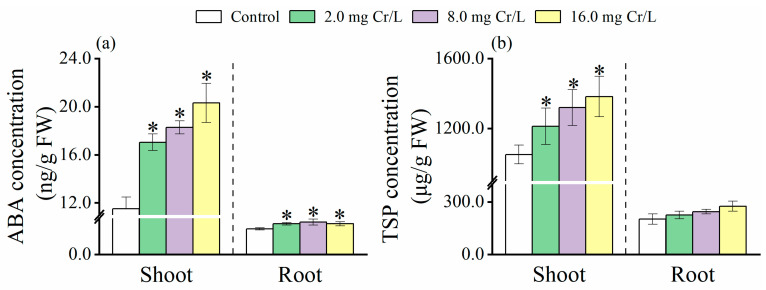
ABA concentration (ng/g FW) (**a**) and TSPs concentration (μg/g FW) (**b**) in roots and shoots of rice seedlings under Cr(VI) stress. The control is 0.0 mg Cr/L. Values are the mean of four independent biological replicates ± standard deviation. The asterisk (*) refers to the significant difference between the treatments and the control.

The ABA concentration in shoots (11.5 ± 0.98 ng/g FW) of the control seedlings was markedly higher (*p* < 0.05) than in the roots (2.12 ± 0.10 ng/g FW) (Figure 2a). On the other side, Cr(VI) treatments induced ABA levels in both shoots and roots, with a more pronounced increase in the shoots. A correlation between Cr accumulation and ABA concentration was observed in both the shoots and roots of Cr(VI)-treated seedlings.

### 3.4. Changes in TSPs

Total soluble phenolics in shoots from both control and treated seedlings were significantly higher (*p* < 0.05) than in roots (Figure 2b). The application of Cr(VI) caused a dose-dependent increase in TSPs. At a concentration of 8.0 mg/L or higher, Cr(VI) significantly increased TSP levels in both roots and shoots (*p* < 0.05) compared to the control.

### 3.5. Changes in Arg, Orn and Met

**Figure 3:**
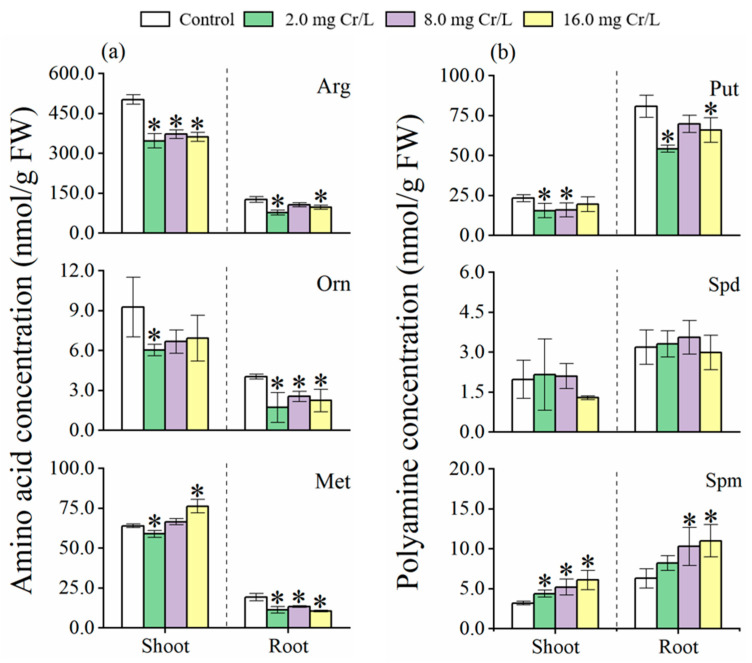
The concentration of Arg, Orn and Met (nmol/g FW) (**a**) and the concentration of Put, Spd and Spm (nmol/g FW) (**b**) in the roots and shoots of rice seedlings under Cr(VI) stress. The control is 0.0 mg Cr/L. Values are the mean of four independent biological replicates ± standard deviation. The asterisk (*) refers to the significant difference between the treatments and the control.

The levels of Arg, Orn, and Met in shoots (Arg: 502.4 ± 17.9 nmol/g FW; Orn: 9.27 ± 2.26 nmol/g FW; Met: 64.0 ± 1.08 nmol/g FW) from the control group were remarkably higher (*p* < 0.05) than in roots (Arg: 126.2 ± 10.5 nmol/g FW; Orn: 4.04 ± 0.18 nmol/g FW; Met: 19.2 ± 2.28 nmol/g FW) (Figure 3a). Cr(VI) application resulted in a decrease in Arg, Orn, and Met levels in rice plants. However, different Cr(VI) concentrations did not affect the concentration of these amino acids in the shoots (Arg: 360.6 ± 12.5 nmol/g FW; Orn: 6.55 ± 0.46 nmol/g FW; Met: 67.3 ± 8.73 nmol/g FW, no = 3) or roots (Arg: 94.0 ± 14.6 nmol/g FW; Orn: 2.17 ± 0.42 nmol/g FW; Met: 11.8 ± 1.49 nmol/g FW, no = 3) of Cr(VI)-treated rice seedlings.

### 3.6. Changes in PAs Compounds

Cr(VI) treatment caused a decrease in Put levels in both shoots (mean: 17.0 ± 2.12 nmol/g FW, no = 3) and roots (mean: 63.4 ± 8.08 nmol/g FW, no = 3) of rice plants compared with the control (shoots: 23.3 ± 2.28 nmol/g FW; roots: 80.9 ± 6.91 nmol/g FW), with the effect of Cr(VI) concentrations being negligible (Figure 3b). Additionally, changes in Spd concentration was marginal between the Cr(VI)-treated rice seedling (shoots: 1.85 ± 0.49 nmol/g FW; roots: 3.29 ± 0.29 nmol/g FW; no = 3) and the control (shoots: 1.98 ± 0.72 nmol/g FW; roots: 3.19 ± 0.64 nmol/g FW). However, Spm concentration in shoots (y = 0.16x + 3.67, *R*^2^ = 0.90) and roots (y = 0.27x + 7.19, *R*^2^ = 0.84) showed a linear increase with rising Cr(VI) concentrations. 

### 3.7. Subcellular Localization of Genes

**Figure 4:**
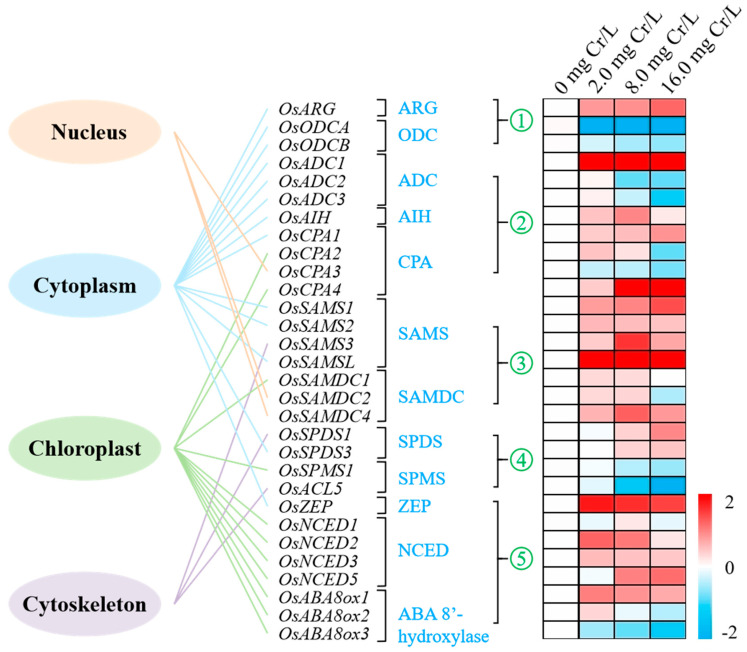
Subcellular localization of the genes associated with the synthesis of PAs and ABA. Heat-map representation of genes detected in the shoots of Cr(VI)-treated rice seedlings (red color refers to up-regulation, whereas blue color represents down-regulation. Heat-map data were taken from the PCR test and shown as log_2_ relative gene expression levels.

An analysis of the subcellular localization of the selected genes is shown in Figure 4. The 30 genes are distributed in the cytoplasm, chloroplast, nucleus, and cytoskeleton, wherein the majority are located in the cytoplasm (*OsARG*, *OsODCA*, *OsODCB*, *OsADC1*, *OsADC2*, *OsADC3*, *OsAIH*, *OsCPA1*, *OsSAMS1*, *OsSAMS2*, *OsSAMSL*, *OsSPDS3*, and *OsZEP*) and chloroplast (*OsCPA2*, *OsCPA4*, *OsSAMDC1*, *OsSPMS1*, *OsNCED1*, *OsNCED2*, *OsNCED3*, *OsNCED5*, *OsABA8ox1*, *OsABA8ox2,* and *OsABA8ox3*). Therefore, only shoots from Cr(VI)-treated seedlings were used for the PCR test.

### 3.8. Gene Expression Profiles

The abundance of genes associated with PMs and ABA synthesis was measured in rice shoots following Cr(VI) treatments (Figure 4). In the first pathway of Put biosynthesis from Arg, *OsARG* was significantly up-regulated (*p* < 0.05), while *OsODCA* and *OsODCB* were down-regulated (*p* < 0.05). In the second pathway of Put biosynthesis from Arg, *OsADC1*, *OsAIH*, *OsCPA1*, and *OsCPA4* were significantly up-regulated (*p* < 0.05) whereas *OsADC2*, *OsADC3*, *OsCPA2,* and *OsCPA3* showed down-regulation (*p* < 0.05) at 8.0 mg/L Cr(VI) or higher. In the Met pathway of Spd and Spm biosynthesis, most genes (*OsSMADC4*, *OsSAMS1*, *OsSAMS2*, *OsSAMS3*, and *OsSAMSL*) were up-regulated, while *OsSMADC1* and *OsSMADC2* showed minimal changes compared with the control (*p* > 0.05). Interestingly, the genes involved in the internal conversion from Put to Spd to Spm displayed inconsistent expression: *OsSPDS1* and *OsSPDS2* encoding SPDS were significantly up-regulated at 8.0 mg/L Cr(VI) or higher, whereas *OsSPMS1* and *OsACL5* were significantly down-regulated (*p* < 0.05).

Among the genes associated with ABA synthesis, five genes, i.e., *OsZEP*, *OsNCED2*, *OsNCED3*, *OsNCED5*, and *OsABA8ox1,* were significantly up-regulated (*p* < 0.05), while *OsABA8ox3* were significantly down-regulated (*p* < 0.05). The expression levels of the remaining two genes (*OsNCED1* and *OsABA8ox2*) showed minor (*p* > 0.05) changes. The detailed results of the PCR tests are provided in the Supporting Information, Figure S1.

## 4. Discussion

**Figure 5:**
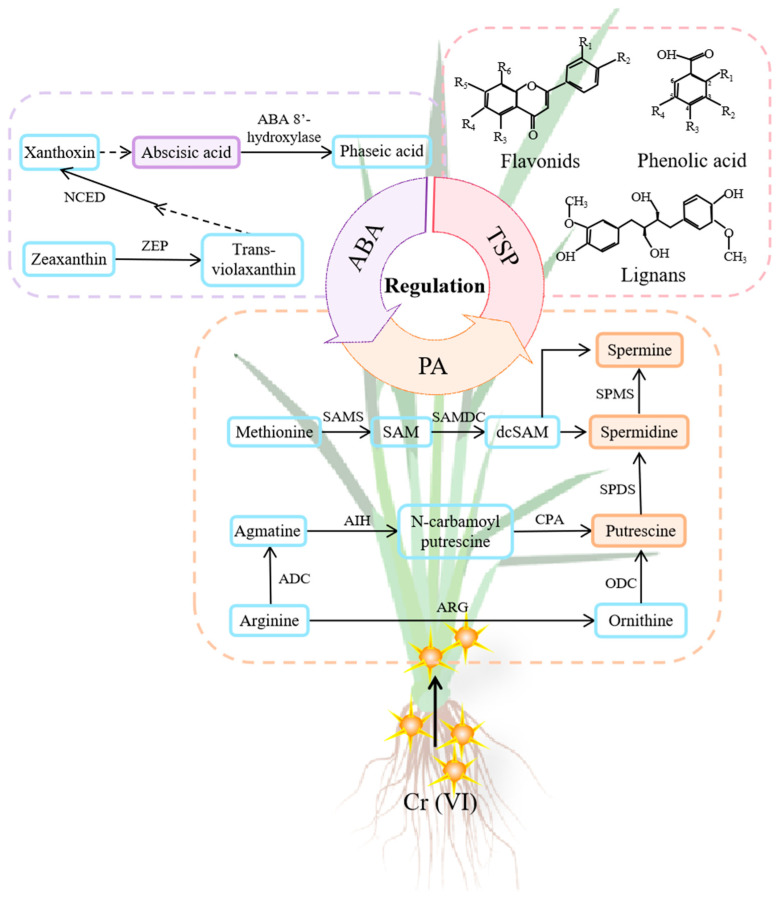
Schematic illustration of pathways involved in synthesis of PAs and ABA.

Putrescine is essential for the biosynthesis pathways of PAs in plants. Figure 5 gives a schematic illustration of the pathways involved in the synthesis of PAs and ABA. The activation of many pathways involved in PA biosynthesis depends on the presence and availability of two synthetic precursors, namely Arg and Met [23,24,26]. The biosynthesis of Put involves two main pathways originating from Arg. In the first biosynthetic pathway, the enzyme ARG initiates the conversion of Arg into Orn, which is further converted into Put in the presence of ODC [23]. The second pathway of Put synthesis also originates from Arg, where Arg is sequentially catalyzed by ADC, AIH, and CPA to produce Put [38]. It is suggested that the latter pathway is the primary synthesis pathway of Put in plants [39]. It is widely recognized that Arg is highly involved in proline (Pro) synthesis through the Orn pathway in plants [40], which can lead to a competition for substrates between Pro synthesis and PA formation. During this study, it was noticed that the levels of Orn in both the shoots and roots of Cr(VI)-treated rice seedlings were markedly lower than those of Arg (*p* < 0.05), indicating that the availability of Orn was a restricting factor for both reactions. Nevertheless, our previous work on Cr(VI)-treated rice plants revealed a rise in Pro levels in the shoots [39], suggesting that the Orn biosynthetic route is not the main route for Put synthesis in rice plants when subjected to Cr(VI) stress. In the current investigation, it was found that the Put levels in the roots and shoots of Cr(VI)-treated rice plants were considerably higher than those of Orn (*p* < 0.05) during the first Put biosynthetic reaction, wherein it is worth noting that the stoichiometry ratio between Orn and Put was 1. This indicates that the production of Put in rice seedlings is mainly associated with the second Put biosynthetic pathway. According to the stoichiometry ratio between Arg and Put in the second Put biosynthetic reaction, 1 mole of Arg can produce 1 mole of Put, assuming the repression of Orn conversion into Put production. Both the control and Cr(VI)-exposed seedlings displayed significantly higher ratios between Arg and Put, indicating that the presence of Arg does not restrict the production of Put from Arg through the second Put synthesis pathway, which aligns with earlier research findings [38,39]. These results offer a significant insight into the intricate processes and interactions involved in Put biosynthesis, as well as the adaption strategies of rice seedlings in response to Cr(VI) stress.

Numerous investigations have documented the detrimental effect of PTE stressors on the metabolism of PAs in plants [41,42,43,44,45]. The relationship between the metabolism of PAs and the reduction in oxidative damage has been extensively examined [46]. In *Phaseolus vulgaris*, the addition of exogenous Spm was found to significantly enhance the activities of enzymes responsible for scavenging ROS, such as SOD, POD, GR, APX, and MDHAR, thereby improving As tolerance by regulating the metabolism of PAs [45]. Another study demonstrated that exogenous Put had a favorable effect on the synthesis of phytochelatins in rice when exposed to Cd stress [26]. Phytochelatin production is an essential process that produces metal-binding proteins in plants, which immobilize and detoxify metal ions [10]. The composition of total and individual PAs in plants is subjected to change, with different proportions of Put, Spd, and Spm [47,48]. This study found that rice plants treated with Cr(VI) had the highest concentration of endogenous Put, followed by Spm, while Spd had the lowest concentration. Exposure to Cr(VI) stress led to a notable decrease in Put concentration, although varying concentrations of Cr(VI) did not affect the levels of Put. However, there was a noticeable and consistent increase in Spm levels as a result of exposure to Cr(VI), but the difference in Spd levels between the control and treated rice seedlings was negligible. These findings suggest that Spd concentration may function as a “gatekeeper” that controls the rate at which Put is converted into Spm. Furthermore, the Spm concentration was markedly higher than Spd, indicating a restricted conversion of Spd into Spm. Nevertheless, it is important to note that there exists an alternate route for the synthesis of Spm from the Met pathway. In the study, it was shown that the expression of genes related to the Met route noticeably increased, suggesting that the branching pathway may have a greater role in the production of Spm in Cr(VI)-treated rice plants. Different studies have reported inconsistent findings regarding the concentration of Put, Spd, and Spm in response to PTE stressors [45]. The variations could be attributed to differences in plant species/cultivars and the specific PTEs used [45,49,50]. For instance, it was recovered that exogenous Spm caused an increase in the levels of Put and Spd in plants, indicating that the reorientation of PAs is an activate strategy used by plants to cope with As stress [45]. Additionally, the application of exogenous Si and Se in As-treated rice plants led to an enhanced production of PAs, including Put, Spd, and Spm, to counteract the harmful effect of As [50].

Plants often experience an increase in naturally occurring levels of endogenous PAs under PTE stresses [45,50], wherein they mostly serve as protective molecules. The homeostasis of PAs is primarily maintained and balanced by their synthesis and oxidative catabolism [51]. Previously, we discovered that oxidative damage in rice plants was directly related to the doses of Cr(VI) used in the treatment, and the internal antioxidant system of rice was effective in removing and scavenging additional ROS through diverse enzymatic reactions [10]. In this analysis, a decrease in Put and Spd levels was observed in Cr(VI)-treated rice seedlings, which contrasts with the findings of previous studies [45,50]. The relationship between the excess or deficiency of total and individual PA concentration and stress tolerance is complex, suggesting that the regulatory role of PAs may be more critical than their protective role in managing stress tolerance [28]. Several studies documented the synergistic or antagonistic interactions between PAs and various phytohormones in plants, indicating positive interactions between PAs and hormones [52,53]. However, in this study, only the concentration of Spm showed a positive correlation with Cr concentration in shoots, while the changes in Put and Spd were independent of the Cr(VI) concentrations. This suggests that PAs may function as signal molecules rather than ROS scavengers in Cr(VI)-treated seedlings. The accumulation of SMs in plants is considered a survival and detoxification strategy to minimize the detrimental effects of HM stresses, regulated by the internal hormone signaling system [16]. In this study, the correlations between TSPs and ABA (y = 0.026x − 14.97, *R*^2^ = 0.97)/Spd (y = 0.008x − 5.71, *R*^2^ = 0.98) in the shoots of rice plants under Cr(VI) treatment were significant (*p* < 0.05). These parameters were also significantly correlated to the accumulation of Cr(VI) in shoots (ABA: y = 0.096x + 12.77, *R*^2^ = 0.87; TSPs: y = 3.87x + 1079.8, *R*^2^ = 0.96; Spd: y = 0.033x + 3.34, *R*^2^ = 0.98), indicating a positive interaction and crosstalk.

Various environmental stresses are known to stimulate secondary metabolism in plants, leading to the accumulation of various SMs in stressed plants [54]. Total soluble phenolics, a class of SMs with a phenol group, are crucial for multiple physiological processes in plants [55]. Several key TSPs, such as flavonoids, anthocyanins, and tannins, have been extensively studied in plants for their role in coping with various environmental stresses [2,55]. Increased concentrations of phenolic compounds are a typical bioindicator in plants under stress [18,56]. Carbon (C) and nitrogen (N) metabolism are fundamental processes involved in plant metabolism [2]. When the C/N balance in plants is disturbed, it can trigger various secondary metabolic pathways in plants under unfavorable growth conditions [54,57]. Furthermore, the homeostasis of PAs in plants through diverse reactions, such as catabolism, biosynthesis, conjugation, interconversion, and transport, serves as a survival strategy to regulate the C/N balance in plants [22]. Consequently, it is probable that there is a positive correlation between PAs and TSPs. This study reveals a noteworthy positive correlation between Spm, TSPs, ABA, gene expression, and tissue Cr in the shoots of rice plants that have been exposed to Cr(VI). These findings highlight the intricate connections between secondary metabolism, PA metabolism, and environmental stress responses. They underscore the importance of comprehending the regulatory processes involved in stress adaptation.

The endogenous concentration of ABA in plants is determined by the dynamic equilibrium between its biosynthesis and catabolism [58,59,60]. Among the enzymes responsible for ABA synthesis, NECD is the key enzyme that regulates the conversion of 9-*cis* epoxycarotenoids to xanthoxin [59]. Arabidopsis research has shown that there is a positive correlation between NECD transcripts and ABA biosynthesis, leading to an increase in ABA levels [61]. In rice, a higher abundance of *OsNCED3*, an NECD isogene, is observed under various types of abiotic stresses such as NaCl, PEG, and H_2_O_2_, suggesting its role in response to abiotic stresses [60]. Our PCR test revealed the significant up-regulation of *OsNCED2*, *OsNCED3*, and *OsNCED5* in rice shoots treated with Cr(VI). Another enzyme involved in ABA regulation is ABA8ox, which is involved in ABA degradation [58]. *OsABAox3*, an isogene of the OsABA8ox gene family, is significantly activated in rice leaves during drought stress, thereby playing a crucial role in enhancing drought tolerance [58]. The ethylene-induced expression of *OsABA8ox1*, driven by ethylene, leads to a reduction in ABA concentration in submerged rice shoots [62]. Furthermore, *OsABA8ox2* and *OsABA8ox3* are involved in the glucose-induced delay of seed germination in rice [59]. During the ongoing inquiry, we noticed different patterns of gene expression for the three isogenes of *OsABA8ox*. Specifically, *OsABA8ox1* was significantly up-regulated, *OsABA8ox3* showed a considerable decrease in expression, and *OsABA8ox2* exhibited minimal changes compared to the control. Additionally, *OsZEP*, a gene implicated in the production of ABA, showed significant up-regulation. The accumulation of ABA detected in rice shoots treated with Cr(VI) indicates that the synthesis of ABA regulated by these genes is highly activated under Cr(VI) stress. This reveals that there a possible coordination between ABA and Spm in increasing the levels of TSPs in rice plants subjected to Cr(VI) stress. In future, additional research utilizing gene modification techniques could offer conclusive evidence on the involvement of specific genes activated in the crosstalk between ABA and Spm to enhance TSP accumulation in Cr(VI)-treated rice plants [63]. This information will be valuable for utilizing agriculture plants to remediate Cr(VI)-contaminated soil in a field trial [64].

## 5. Conclusions

The study investigated the interactions between ABA, Spm, and TSPs within the metabolic network of rice plants subjected to Cr(VI) stress. The accumulation of Cr(VI) in rice tissues led to inhibition of plant growth. The responses of ABA, TSPs, and Spm to Cr(VI) treatment in rice plants were found to be positive. By integrating the analysis of PM-related compounds with PCR testing, it was revealed that the second pathway originating from Arg and the Met pathway were highly activated in the synthesis of Spm. The interconversion between the three species of PMs showed enzyme limitation. These findings suggest that the interplay between ABA and Spm contributes to enhanced TSP accumulation in Cr(VI)-treated rice plants, acting as a survival mechanism to manage Cr(VI) stress. Such findings unveil a crucial understanding of the metabolic dynamics within rice plants under Cr(VI) stress, wherein the interactions between ABA, Spm, and TSPs not only shed light on the plant’s response mechanisms but also highlight a survival strategy in managing the detrimental effects of Cr(VI) accumulation.

## Data Availability

The data is available at Supplementary Materials and the public databases mentioned in the study.

## Supplementary Materials

The following supporting information can be downloaded at: https://www.mdpi.com/article/10.3390/toxics12080577/s1, Figure S1: The relative expression of genes associated with the synthesis of PAs and ABA in rice shoots under Cr(VI). Values are the mean of four independent biological replicates ± standard deviation. The asterisk (*) refers to the significant difference between the treatment and control; Table S1: Genes involved in polyamine metabolism in rice seedlings.
