# Supplementary material for: The Accumulation of Abscisic Acid Increases the Innate Pool of Soluble Phenolics through Polyamine Metabolism in Rice Seedlings under Hexavalent Chromium Stress
Source: Toxics. 2024 Aug 8;12(8):577. doi: 10.3390/toxics12080577 (PMC11359078; doi:10.3390/toxics12080577)
Supplement: Supplementary file 1 [file toxics-12-00577-s001.zip › toxics-3137644-supplementary.pdf]

Supplementary Materials: The Accumulation of Abscisic Acid Increases the Innate Pool of Soluble Phenolics through Polyamine Metabolism in Rice Seedlings under Hexavalent Chromium Stress

Yi Kang, Cheng-Zhi Li, Abid Ullah**, Qing Zhang and Xiao-Zhang Yu**

Measurement of total Cr

After 2 d exposure, the roots of seedlings were washed with deionized water and then divided into root and shoot parts. The digestion solution (4:1 HNO_3-$HClO_4$) was used to digest the oven-dried plant materials (48 hours at 96°C). The concentration of Cr in rice tissues (µg/g DW) was measured with the help of ICA-AES (PerkinElmer Optima 700DV). For the element measurement, Cr at 267.716 nm was used. The detection limits, determined as mean blank plus three times the standard deviation of ten blanks, was 0.07 µg Cr/L.

We calibrated the instrument response and sample preparation variations by adding internal standards. After that, the standard curve was made using the following concentrations: 0, 0.2, 0.5, 1.0, 2.0, 5.0, and 10 .0 mg/L. During the sample measuring process, we also checked the stability of the analytical process using the quality control (QC) samples with known concentrations. The QC results were recorded and we calculated the relative standard deviation (RSD). The RSD from the same QC sample, based on measurements taken 3-4 times, is less than 10%, indicating that the experimental data are reliable.

Measurement of ABA

Extraction and Purification of tissues samples*:* 100.0 mg of the powdery tissue samples was homogenized and extracted for 12 h in 1.0 mL of methanol solution (0.2% formic acid). The process was isolated from light and kept at 4°C. Next, the mixture was centrifuged at 14,000 g for 10 min at 4°C. The supernatant was taken and evaporated with N_2. Subsequently, the residue was resuspended in 0.2 mL of methanol solution (50%, v/v) and then centrifuged at 14,000 g for 10 min at 4°C. The supernatant was collected for ABA analysis.

UPLC-MS analysis: The mobile phase buffer included a combination of buffer A (0.04% formic acid in water) and buffer B (0.04% formic acid in acetonitrile). The injection volume was 4.0 µL at a flow rate of 0.4 µL/min, the automatic sampler temperature was 4°C, and the column temperature was 45°C. The linear gradient was as follows: 2%→98% B (v/v) for 10 min; 98%→2% B (v/v) to 10.1 min; and hold at 2% B to 13 min.

The MS system (5500 QTRAP System, AB SCIEX) equipped with an electrospray ionization source was operated in negative ionization and multiple reaction monitoring modes. The running conditions of 5500 QTRAP were as follows: source temperature, 500 °C; ion source gas 1, 45 psi; ion source gas 2, 45 psi; curtain gas, 30 psi; and ion spray voltage floating, -4500 V.

Measurement of total soluble phenolics

Soluble phenolics were determined by the Folin–Ciocalteu method. Briefly, 500 µL of supernatant was mixed with 300 µL of 1 N Folin and Ciocalteau's phenol reagent. The mixture in the tube was placed on a rotating tube holder at 25 °C for 5 min. And then, the solution was reacted with 400 µL of 1 M Na_2CO_3 and kept at 25 °C for

10 min. Finally, the solution was made up to 5.0 mL with deionized water and allowed to stand for 1h at 25 °C. In order to avoid photo-oxidation, all tubes were covered with aluminum foil. The absorbance was measured at 725 nm. The blank contained 500 µL 80% methanol plus Na_2CO_3 and the Folin and Ciocalteau reagent. The content of TSPs was expressed in µg/g FW, based on the standard curve of gallic acid as follows.

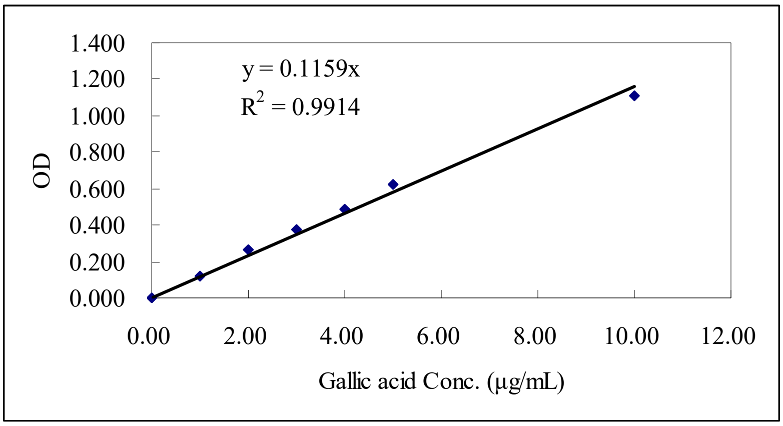

Measurement of polyamine-related compounds

Preparation of standard solutions and samples: Standard solution series of 0.1, 0.5, 1.0, 2.5, 5.0, 10.0, 25.0, 50.0, and 100.0 ng/L were prepared. The external standards were prepared in ultrapure water with 0.1% hydrochloric acid aqueous solution. After exposure, rice tissue samples were respectively collected from treated and controlled seedlings and then preserved in liquid nitrogen before freeze-drying. Moreover, 50 mg samples were weighed after liquid nitrogen grinding. Furthermore, 1 mL precooling methanol/acetonitrile/water (2:2:1, v/v/v) was to each sample, and the samples were subjected to vortex blending (US, MET VXT-200), a low-temperature ultrasound for 30 min, and incubation at 20°C for 1 h for the deposition of proteins. A high-speed low-temperature centrifuge (Eppendorf 5430R) was set to 4°C and 14000 RCF for 20 min, and then the supernatant was extracted, and vacuum-drying was performed. For mass spectrometry detection, 100 µL acetonitrile/water solution (1:1, v/v) was added for resolution, and the supernatant was centrifuged at 4°C and 14000 RCF for 20 min. The supernatant was filtered through 0.22 µm membrane and diluted twice before sample analysis.

Instrument conditions: The chromatographic separation and identification processes were carried out by the Agilent 1290 Infinity Acquity UPLC system. UHPLC separation was performed with a Zic-HILIC 3.5µm, 2.1 mm×150 mm chromatographic column. The gradient mobile phases consisted of acetonitrile (eluent A) and 25 mM ammonium acetate aqueous solution adjusted to pH 6.8 with acetic acid (eluent B). The gradient elution was conducted with the following conditions: 0–2 min, 2% A; 2– 6 min, 2–10% A; 6–10 min, 10% A; 10–16 min, 10–20% A; 16–22 min, 20% A; 22–25 min, 20–30% A; 25–26 min, 30% A; 26–29 min, 30–60% A; 29–33 min, 60% A. This was followed by the washing and reconditioning of the column. The flow rate was 0.25 mL/min, the column temperature was maintained at 40◦C, and the injection volume was 2 µL. A 5500 QTRAP mass spectrometer (AB SCIEX) was used for mass spectrometry in the positive ion mode. The collection mode is the multi-response monitoring (MRM) mode. The 5500 QTRAP ESI source conditions were as follows: temperature: 500°C; ion source gas 1 (Gas1): 40; ion source gas 2 (Gas2): 40; curtain gas (CUR): 30; ion spray voltage floating (ISVF): 5500 V; and the MRM mode was used to detect the ion pairs to be measured.

Table S1. Genes involved in polyamine metabolism in rice seedlings.

Gene	Locus identifier	Primer sequences (5′-3′)	Amplicon size (bp)
OsARG	LOC_Os04g01590	CATTCTTCACCTTGACGCACAT<br>GCCTCCTAGCATAACCTCCTTC	110
OsODCA	LOC_Os09g37120	GGCGTCAAGGACAAGAAGG<br>GAGTCGTAGGTGGTGAGGTT	398
OsODCB	LOC_Os02g28110	GAGGTGGTGAGGGGTTATCAG<br>TGGAGAACCCGTTGAAGTCTG	112
OsADC1	LOC_Os06g04070	CAATGCCGTACCTGTCGTT<br>CACTCGTCGTCATCCTCCT	121
OsADC2	LOC_Os04g01690	GCACCACTCCATGATCATCC<br>TTCGCCAGCTTGTACATCTC	216
OsADC3	LOC_Os08g33620	CAATCATCCCAATCCAGTGCC<br>ATTGCGCCGAACGCTTTG	295
OsAIH	LOC_Os04g39210	GGACGGCAGATAGAAGTTGTG<br>TTCCACCATCACCACCTCAT	252
OsCPA1	LOC_Os02g33080	GCGGAGAGGTTGATAAGAGAAG<br>CCTGGTCCATCTGGAATATGTG	302
OsCPA2	LOC_Os03g07910	GCCTTCTCACTCCTCACCT<br>CGATGTTGCGAGCCTTGT	238
OsCPA3	LOC_Os06g10420	TCAACACTTGCTGCGTGATCGG<br>TCCAATGCCAATCCGTCCAACA	173
OsCPA4	LOC_Os12g31830	TTCTCATTCATAGGCTCCAAGG<br>CTCCAAGAGACAACCACATACT	121
OsSAMS1	LOC_Os05g04510	GCCGCACTTGATACCTTCC<br>GTCACCAGCACCAATCTCC	372
OsSAMS2	LOC_Os01g22010	TGATGGTGTTCGGCGAGAT<br>TGGAGATGAGGACGGTGTG	432
OsSAMS3	LOC_Os01g18860	TGCTTGATGCGTGCCTTG<br>CCACTGTCACTTGCGTCTT	444
OsSAMSL	LOC_Os07g29440	GTTCCTCTTCACCTCCGAGTC<br>CGATGTTGACGAGCACCTTG	281
OsSAMDC1	LOC_Os04g42095	CGACGCCTCTACTCTTGCTTAT<br>CATTCTCCTCCAACGCATCCT	359
OsSAMDC2	LOC_Os02g39795	CTCTGTTGCGGTTACCATCTT<br>CCTCTGTGCCATCTATCTCCT	278
OsSAMDC4	LOC_Os04g42095	CTGTTACTCCTGGTGCTGTGT<br>TTCTTCTTCGCCGCCTTCTC	127
OsSPDS1	LOC_Os07g22600	CGGCAAGGTGCTTGTGCTGGAT<br>GGCCACGCCATCTCCAATGTGT	298
OsSPDS3	LOC_Os02g15550	TGGTTATTGGAGGTGGTGATGG<br>GAAGCCGAACACGAGGATCTT	162
OsSPMS1	LOC_Os06g33710	GCCTGGACAAGCGTTCCTACCT<br>GCAGCCCGATGAACCTCTGAAT	173

OsACL5	LOC_Os02g14190	GCTCTGCTACGCTCTCAACA<br>GAACTCGTCCACCTCTGTACTC	136
Os*ZEP*	LOC_Os04g37619	CGTGCGGTTGGTGATGATGCT<br>GCACCGACATCTGAGGAGACGA	299
OsNCED1	LOC_Os02g47510	CACTGCTCGGTCACTCACTC<br>GCTACCAACTGCTCGTCCTC	221
OsNCED2	LOC_Os12g24800	GAGGCTGATGGAGAGGAGATTG<br>TGGATGACACCGAACTCAAGG	176
OsNCED3	LOC_Os03g44380	GACTGCTTCTGCTTCCACCTCT<br>TCGTCGGACTCGTTGAAGATGG	113
OsNCED5	LOC_Os12g42280	TGCTTCTGCTTCCACCTGTG<br>GTGTTCGTCCGACTCGTTGA	114
OsABA8ox1	LOC_Os02g47470	GCTACCTGACGCTGGAGAAG<br>CGCCGAGGAACTTGACCAT	282
OsABA8ox2	LOC_Os08g36860	GCGAGATTGGCAGAAGAAGAAG<br>TGCTTGGAGGCGAAGAAGAC	120
OsABA8ox3	LOC_Os09g28390	GATGGAAGGTGATGCCGCTCTT<br>CCTCGTCGCTGGAACCAACAAT	237
OsGAPDH1	LOC_Os08g03290.1	F-GACAGCAGGTCGAGCATCTTC<br>R-CAGGCGACAAGCTTGACAAAG	74

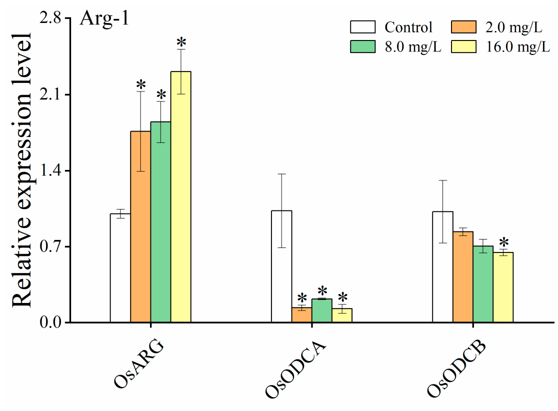

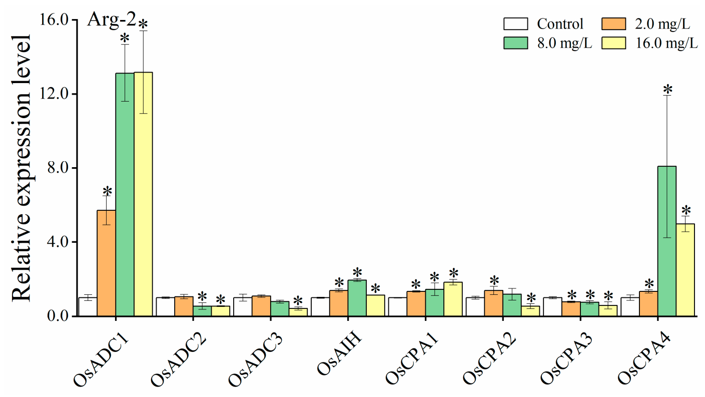

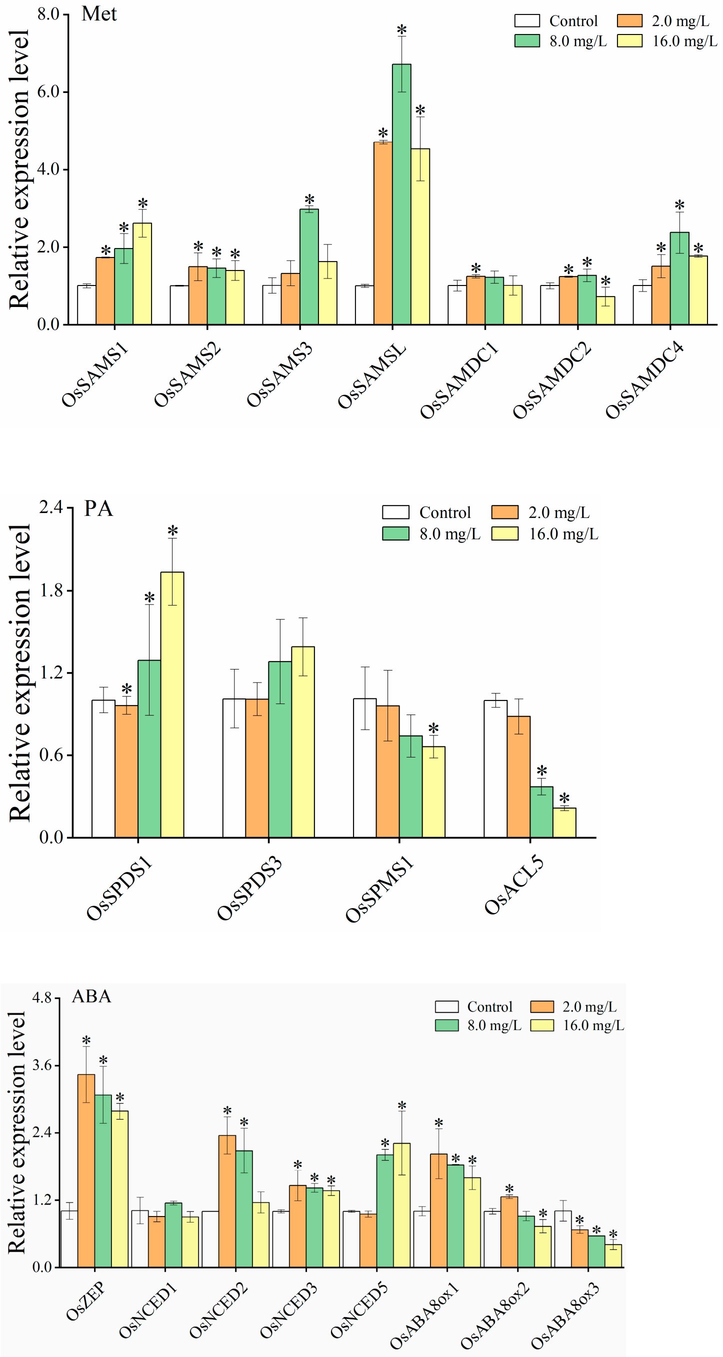

Figure S1: The relative expression of genes associated with the synthesis of PAs and ABA in rice shoots under Cr(VI). Values are the mean of four independent biological replicates ± standard deviation. The asterisk (*) refers to the significant difference between the treatment and control.
